# Experiences of Families Caring for Children with Newborn Screening-Related Conditions: Implications for the Expansion of Genomics in Population-Based Neonatal Public Health Programs

**DOI:** 10.3390/ijns8020035

**Published:** 2022-05-23

**Authors:** Lynn Bush, Hannah Davidson, Shani Gelles, Dawn Lea, Laura M. Koehly

**Affiliations:** 1 Division of Genetics and Genomics, Boston Children’s Hospital and Center for Bioethics, Harvard Medical School, Boston, MA 02115, USA; lynn.bush@childrens.harvard.edu; 2 National Human Genome Research Institute, Bethesda, MD 20892, USA; hannah.davidson@nih.gov (H.D.); shani.gelles@nih.gov (S.G.); dawn.lea@nih.gov (D.L.)

**Keywords:** newborn screening sequencing, newborn genomics, ethics, psychosocial, pediatric rare disease

## Abstract

With the expansion of newborn screening conditions globally and the increased use of genomic technologies for early detection, there is a need for ethically nuanced policies to guide the future integration of ever-more comprehensive genomics into population-based newborn screening programs. In the current paper, we consider the lived experiences of 169 family caregivers caring for 77 children with NBS-related conditions to identify lessons learned that can inform policy and practice related to population-based newborn screening using genomic technologies. Based on caregiver narratives obtained through in-depth interviews, we identify themes characterizing these families’ diagnostic odyssey continuum, which fall within two domains: (1) medical management implications of a child diagnosed with an NBS-related condition and (2) psychological implications of a child diagnosed with an NBS-related condition. For Domain 1, family caregivers’ experiences point to the need for educational resources for both health care professionals that serve children with NBS-related conditions and their families; empowerment programs for family caregivers; training for providers in patient-centered communication; and access to multi-disciplinary specialists. For Domain 2, caregivers’ experiences suggest a need for access to continuous, long-term counseling resources; patient navigator resources; and peer support programs. These lessons learned can inform policy recommendations for the benefit of the child, the family, the healthcare system, and society.

## 1. Introduction

As the expansion of population-based newborn screening (NBS) accelerates globally with the increasing integration of genomic technologies, the anticipation of potential implications arising from opportunities and challenges is essential to optimize benefits and mitigate harms [1,2,3,4,5,6,7,8,9,10,11]. This public health endeavor will have a significant influence and impact on many stakeholders [12,13,14], not the least of whom are the infants receiving a “true-positive” finding and their families. Thus, it is critical to consider the lived experiences of family caregivers who have been navigating the complex landscape of their children’s rare conditions that are currently or potentially identifiable through NBS programs. We collectively refer to these disorders as “newborn screening-related conditions,” reflecting gene and disease inclusion on the US Recommended Uniform Screening Panel Core and Secondary, NBSTRN-CR Candidates, NC-NEXUS, and others published by experts (i.e., mitochondrial disorders) [15,16,17,18].

We also extend the concept of a “diagnostic odyssey” to consider the broader journey that caregivers of children with rare NBS-related conditions can experience in their attempt to understand and manage the uncertain spectrum and evolving course of the newborn’s reported screen, presumptive diagnosis, and confirmatory diagnostic results. For even a molecular finding is often not the end of their diagnostic odyssey. We refer to this broader journey as the “diagnostic odyssey *continuum*” (see Bush, Al-Hertani, Bodamer in this Special Issue [19]). Important to this concept is the under-appreciated fact that NBS is a process, not simply a test, and will likely remain a process whether as a “screening” test through current NBS platforms or a genomic “diagnostic” test from the outset. Either way, public health newborn screening programs aim to presymptomatically identify select conditions for which a confirmatory “diagnosis” may be sought, and often diagnosis is an evolving process. Typically, findings are initially communicated in the beginning of the first year of life with the intent that through early identification, negative outcomes for the infant can be averted or mitigated, thereby reducing morbidity and mortality rates for all infants [20,21]. In doing so, beneficence and non-maleficence can be actualized and equitable [9]. However, early signs and symptoms may be missed, the presumptive diagnosis may be a misdiagnosis, or the confirmatory diagnosis may not fully capture the child’s condition due to significant variability, resulting in a ‘label’ that lacks precision. In such cases, families are often on a journey that extends well beyond the initial diagnosis, a journey that continues until a fully realized syndrome is manifest, more precisely identified, and outcomes understood.

Importantly, the heterogeneity in phenotype and genotype-phenotype correlations of many NBS-related conditions (current and proposed) results in prognostic uncertainty which can impact the timespan of the diagnostic odyssey continuum. Long-term follow-up of NBS is key [22,23], yet notably lacking, and could improve understanding of a rare condition’s natural history [24]. This is particularly salient as NBS expansion with genomics accelerates and amplifies the need for shared decision-making [25,26] in care management given the level of uncertainty that will accompany the greater magnitude of conditions and carrier statuses identified. While recent studies report opportunities, challenges, and limitations with augmenting, supplementing, and secondary tier use of genomic approaches to characterize this variation [6,27,28,29,30,31], and some professionals are vocal proponents or opponents of primary comprehensive sequencing as part of NBS [32], it is important to seek the voices of caregivers and consider the lived experiences of families who are already caring for children with NBS-related conditions. Such perspectives can contribute to ethically nuanced policy.

From an ethical perspective, the “best interest of the child” [33,34] and the “harm principle” [35] are beacons that guide NBS practices worldwide. However, there is controversy [36] about whether NBS criteria should go beyond the classic 1968 Wilson Jungner [37] World Health Organization (WHO) framework to a broader approach with consideration of parent/family interests and future planning. Andermann and colleagues [38] posited one such screening criteria (also for the WHO) four decades later that they subtitle “for the genomics era” (not necessarily WGS) and more closely parallels contemporary practice involving children. Their framework includes weighing benefits for family interests while not neglecting the child’s interests, and is applicable whether sequencing is primary, secondary, confirmatory, or supplemental. Further controversy surrounding inclusion criteria arises from the degree of comprehensiveness, and their potential benefits and harms [32,39,40]. Some commentators note that the incorporation of a genotype-first approach in NBS for an expanding range of conditions may no longer constitute a newborn “screen” and potentially jeopardize a valuable public health program with some parents opting out of consenting. Others argue a more comprehensive genotype-first approach in NBS can shift a learning health care system [41] further from a reactive model to a proactive model, and in turn, improve equitable access to diagnostics and potentially make more actionable diagnoses pre-symptomatically. Doing so, even with less comprehensive panels, may then result in more equitable access to treatments as well as potentially provide greater opportunity for families to make future plans for the child and other family members [42].

With these potential opportunities in mind, and cognizant that many ethical concerns co-exist (see others in this Special Issue), we consider caregiver narratives as they describe their journey through the diagnostic odyssey continuum. The psychosocial implications of their experiences, powerfully articulated from their voices, are particularly timely to help guide an ethically minded approach that is beneficial to the child and that eases the burden for families and minimizes harms in the context of sequencing informed NBS. What we learn from these implications derived through interviews most certainly has ethical implications in the accelerating implementation of genomics in public health NBS programs. Thus, the integration of caregivers’ perspectives into policies that guide genomic expansion within population-based NBS has significant value and fosters a family-centered approach to informed decision-making. Moreover, policies that arise from such work can aid in identifying what is required to manage expectations of phenotypes, medical/educational/social needs, outcomes, quality of life, and caregiving responsibilities, while also maximizing benefits and minimizing harm for the family and the child.

The framework of this study is in contrast to the preponderance of research that investigates the ethical and psychological implications of parents receiving false-positive newborn screening results, carrier status, or true positives of single conditions, which typically utilize surveys, focus groups, questionnaires, or interviews that directly inquire about the NBS process. Notably, for previous interview-based NBS studies, relatively small samples were typically engaged. In the current paper, data were obtained from a relatively large sample of caregivers participating in interviews that did not probe specifically about NBS. This study also diverges from most rare disease qualitative research similarly focused on the psychosocial experiences of families in that those studies tend not to then use their findings to generate NBS ethical considerations. In the current paper, we characterize the experiences of caregivers whose children’s illnesses are NBS-related conditions and examine their experiences and perspectives in the context of a diagnostic odyssey continuum. We discuss the implications of caregivers’ experiences and describe “lessons learned” for guiding ethically nuanced population-based newborn screening expansion involving sequencing in public health programs. Using qualitative analysis of open-ended interviews from a relatively large sample of individuals caring for children with NBS-related conditions, we consider the following questions:

- How does the presumptive diagnosis of a potentially NBS-identifiable condition impact these families’ accounts of their diagnostic odyssey?
- How do these caregivers describe the evolution and communication of diagnosis, prognosis, variation, and uncertainty as they navigate the process of understanding the child’s condition?
- What meanings and themes arise when these caregivers discuss their experiences with caregiving regarding their past and present reality in the context of a diagnostic odyssey continuum, as well as its impact on future planning?
- What lessons can be learned from the themes identified from these caregiver perspectives to optimize ethically nuanced NBS expansion augmented with sequencing?

The intent of this empirical research is to build upon prior and current efforts in the literature and contribute to the evidence base for developing timely recommendations for guiding the future integration of more comprehensive genomics into NBS. Importantly, in line with ethical principles and a family-centered approach, this work will also inform the identification of caregivers’ needs in order to make informed decisions and provide resources that will better support caregivers in their care roles as well as enhance shared decision making. Thus, the lessons learned through caregivers’ experiences can help guide policy and practice as it relates to population-based newborn screening with increasing genomic technology in public health programs.

## 2. Materials and Methods

### 2.1. Procedures

Data come from the ‘Inherited diseases, caregiving, and social networks’ protocol (Clinical Trials Identifier NCT01498263), a mixed-methods cross-sectional study that focuses on family caregiving in the context of several rare genetic diseases and undiagnosed conditions. The study was approved by the Institutional Review Boards of the National Human Genome Research Institute and the National Institutes of Health. Participants were recruited through ongoing clinical studies of persons affected by inherited metabolic and mitochondrial conditions and undiagnosed diseases; family meetings and advocacy groups for such rare genetic conditions; and referrals from participating family members.

Following informed consent, eligible participants completed an online survey and semi-structured interview either by telephone or in person. Characteristics of the participant and child were ascertained through both survey and interview. Of note, “child” is used inclusive of those who reached adulthood by the time of the interview. Participant characteristics include age, sex, race/ethnicity, socio-economic status, and kinship tie to the child. Characteristics of the child include age, sex, and diagnosis; in addition, caregivers completed the Children’s Habilitation Assessment Tool (CHAT) [43] which provides context for the severity of the child’s condition.

Participants also completed a semi-structured interview where they were asked a series of open-ended interview questions assessing their physical and emotional health, experiences with caregiver ‘burnout’, and the impact of caregiving on family functioning. The semi-structured interview guide (see Supplementary Materials) was derived from a previously published study investigating caregiver health in the context of caring for children with genetic syndromes, learning and attentional disorders, and neurological disorders [44]. All interviews were transcribed verbatim; personally identifiable information of participants, the child, and any family members discussed within the interview was redacted prior to analysis. The current report focuses on a subset of participants who are family caregivers of children with a newborn screening-related condition (those currently or potentially identifiable through NBS), specifically 169 participants from 77 families.

### 2.2. Sample Characteristics

Family caregivers of children with NBS-related conditions were, on average, 42 years of age, and approximately 70% of these caregivers were parents of these children. Two-thirds self-identified as female (66.9%) and the majority reported being married (82.2%). Slightly more than 10% had some high school or a diploma, while just under a quarter had an associate degree or technical training; most caregivers completed a bachelor’s degree (42.6%), and one-fifth had post-graduate training. Slightly more than twenty percent of caregivers reported an annual household income of USD 50,000 or less, while about forty percent reported an annual household income of at least USD 100,000. The majority of caregivers identified as non-Hispanic or Latino (90%) and White (84%) (see Table 1).

The children with NBS-related conditions were, on average, 10 years of age; a few were over 18 years of age when their family caregiver was interviewed. About a third of the children were female (36.4%) and the majority (80.5%) were diagnosed with an organic acidemia. Based on the CHAT [43], care needs were largely in the life skills domain, with disability scores for social skills and behavior falling in the mid to lower ranges, respectively (see Table 1). Higher scores are indicative of greater support required for activities of daily living in the respective domain.

### 2.3. Analysis

Families who referenced caring for children with NBS-related disorders were identified after multiple readings of the open-ended transcripts (LB). NVivo 12 Plus Version 12.6.0.959 (64 bit), QSR International, Melbourne, Australia [45], a qualitative data analysis software program, was used to facilitate coding and analysis. A preliminary iterative codebook was developed after an initial round of coding (SG and HD); 52 participant transcripts, from 25 families, were double coded to identify recurrent themes related to the proposed research questions, organized by overarching themes and more specific motifs. Inter-rater reliability was assessed, and these preliminarily coded transcripts were reviewed to identify additional codes and revise initial codes. The remaining transcripts (117 participants from 52 families) were randomly divided between two coders (SG and HD) for single coding according to the established codebook. Coders met to further refine existing codes and identify sub-codes throughout this second phase of analysis. After reaching saturation, codes were shared with the research team at-large (SG, HD, LK, DL, LB). Coders (SG, HD, LK, LB) grouped salient codes into broader thematic categories in accordance with research questions. Aspects of grounded theory and inductive qualitative analysis were used to help coders avoid preconceptions and to find unanticipated themes and commonalities within the corpus of data [46,47]. Participant unique identification numbers follow quotes used to illustrate identified themes; the first three digits of each identification number denote the family, followed by three digits that denote the family caregiver.

## 3. Results

### 3.1. Domain 1. Medical Management Implications of a Child Diagnosed with NBS-related Condition: “there’s so much, still, that’s unknown” (747000)

Caregiver responses highlighted the multi-faceted challenges arising from their experiences of diagnoses for rare diseases. They describe their experiences as an odyssey that often move families into a caregiving role filled with uncertainty. One component of this uncertainty was expressed as being related to how much is unknown about the rare conditions affecting the child and the limited understanding of such conditions within the medical community. These unknowns made their challenging caregiving roles ever more complex. Family caregivers were acutely aware of how much there is still to learn, for both themselves and medical professionals:

“*There was a lot of unknowns, a lot of unknowns. What is his condition? And what’s it all going to mean?*” (747001)

“*-- we had a doctor tell us one time that if it has hooves it’s probably a horse and not a zebra. It’s like, well, we were the zebra. Like [child], you know, didn’t have reflux, he had a metabolic disease…*” (119000)

“*We were really worried initially about [the child]’s, like, physical health. Like, there wasn’t very much research on his disorder and what there was, kind of, was very dire and like, ‘Oh, he could have these medical crises at any second.’*” (702000)

Many of these caregivers also expressed that in addition to the rarity of these conditions, the phenotypic variability within conditions compounded their challenges with ongoing uncertainties. A medically named diagnosis does not capture the lived experience or prognosis of the individual child and family.

“*We just don’t even have any clue what the outcome’s going to be like in this… The spectrum is so wide.*” (116001)

“*When you read things online, it can be, like, sort of, like, worst-case scenarios… when I first read about [condition] when he first got diagnosed, like, this could be true, but this hasn’t turned out to be true for him or the majority of other patients we know.*” (126004)

The rarity of such conditions, and phenotypic variation within, reportedly shaped these families’ diagnostic odyssey continuum—that is, how they inform themselves about the child’s condition following a presumptive diagnosis and subsequent modifications to the diagnosis, act as patient advocates, interface with the medical community, and approach treatment-related decisions.

#### 3.1.1. “And then the next day we heard, ‘[condition]’, and it has just been a whirlwind since then.” (140000)

While the odyssey from the unknown to a “screen” to a “diagnosis” for current NBS conditions typically begins at birth with the heelstick, for most of our participants the diagnostic odyssey continuum did not end at the point of the first reported finding.

“*…neurologist at the hospital—he said something I’ll never forget… ‘as soon as something like this happens, all they [sic parents] think about is, ‘Is my baby going to die? …You just need to be aware of where your mind goes when you look at your child. Especially when they’re at the doctor, or you’re getting new and different news about the diagnosis.’ And our geneticist in [city] told us that he—because I asked him point-blank, ‘Okay, so what’s the lifespan for a kid who has this?’ At the time, we didn’t know his mutation. He told us maybe 11 or 12 years old.*” (112000)

“*-- when he was in the hospital as a brand-new newborn,…. I said, ‘Man, let’s keep things in perspective. The baby next to us has to get a liver transplant,’ and that, like, things were much worse for them than they were for us. And then, eight months later, there we were, getting a liver transplant.*” (126004)

As described, in some cases, the child’s clinician incorrectly considered the initial results to be a ‘false positive’, *“It’s too rare of a disease. He doesn’t have this disease. This was a false positive.”* (119000), extending experiences of uncertainty as families awaited a conclusive result. For several, the child experienced a medical crisis before results were disclosed.

“*Well, it was caught in the screening. However, I think it took too long, because she was 7 days old when we got the call that something was abnormal on the screening, but she was already sick by then, and they—all they said was, ‘Abnormal.’ So, we took her to the pediatrician. They did more bloodwork and, I think, got urine and stool samples, something like that, and sent it off and told us -- … told us to keep feeding her. Well—but it was the next day that we were in the ER…I mean, it was newborn screening-detected, so that’s great. That probably saved her life. She might have been a SIDS baby otherwise. But…knowing about it sooner would—could have possibly prevented the metabolic crisis.*” (140000)

“*…but then his dad was like, “We’ll just wait until the doctor contacts us, or like contacts us, and like, you know, they’ll handle it”, but they didn’t, and then he had like seizures and he had to keep him in NICU.*” (148000)

Given caregivers’ limited understanding of such rare diseases, there is a reliance on the medical team, who may also have limited understanding of and limited experience with these rare conditions. Yet, healthcare professionals don’t always recognize, or share, that the confirmation testing following an initial screen and consultation process can be lengthy. Such delays not only can have significant medical implications for the child, but they also have the potential to impact patient–provider relationships moving forward. This time period is pivotal in shaping how caregivers interface with the medical community.

“*-- all of the initial testing where…we were kind of naive and, like, we didn’t know, like, of course we were going to go along with what the doctor said, and like, it’s kind of like they tested the same thing four times just to make sure.*” (745000)

“*Yeah, well, that’s the thing. Like, I don’t feel like—now, in our first year it was a lot of new information thrown at us all the time. He had 86 doctor’s appointments in 10 months.*” (401000)

#### 3.1.2. “-- just make sure you learn as much as you can” (150001)

As these caregivers entered the world of rare diseases, they speak about learning and processing information that encompasses both the intricacies of their child’s condition, as well as the nuances of the health care system. Many considered developing a literacy in this world as being paramount to providing effective care for their child. Said one parent:

“*It’s pretty overwhelming. Like, it’s pretty, like wow, like this is intense, you know, to not only be in—like to be the one that’s, you know, helping to keep these kids alive, but like to like, have all this medical stuff that you have to do along with it. So, it’s definitely a learning curve, like you learn a lot of information that no one else understands, unless you run into someone who understands it.*” (110000)

Caregivers describe a vast range of resources used to build their knowledge base, ranging from the more professional and academic (including keeping up to date on emergent research and primary literature) to the more informal, such as parent Facebook groups. They spoke of the necessity of building this knowledge base as being rooted in the rarity of the conditions themselves:

“*Well, I was the one up on the internet, you know, up on the computers trying to research—he was detected through newborn screening that [child] had [condition] …And no one at the hospital where he was born had—knew what this was… So, even though we were in a big city hospital with all these great doctors, no one had heard of this…condition.*” (747000)

However, the available sources for information gathering can yield mixed results: caregivers note both the necessity to keep up to date with information, and also acknowledge that it is imperative to “*take the things you read with a grain of salt*” (126004) since some of the sources were outdated or did not address phenotypic variability within the condition.

#### 3.1.3. “like, become informed, because you’re going to have to be an advocate when your child has a rare disease” (126004)

As a result of extensive information gathering, caregivers stressed the need to become expert advocates for their child, ever cognizant of the gaps between the care system they and the patient must navigate and the state of the science on their child’s condition:

“*Recognize that if you want your child to have the best care, you’re going to have to really do that research and, like, stick up for things, because many doctors have only seen one or two cases of this in their career.*” (126004)

Many acknowledge that stepping into the role of expert advocate requires caregivers to develop considerable information-gathering and organizational skills:

“*-- we take the time to go see a doctor four to five hours away, we have a list of questions… then we have time for other questions that may arise spontaneously… But I think the organization and the openness in conversation and planning ahead has helped.*” (112000)

#### 3.1.4. “finding those right doctors … is the most important thing” (116001)

For many of these families, an imperative action in the initial time frame and long-term is building an effective, knowledgeable, and cohesive care team. In addition to gathering information and advocating for their child, caregivers also need to identify health care providers that have the appropriate expertise and patient-centered communication skills. For many, locating such experts is challenging, often requiring trial and error and out-of-state travel:

“*I went to [advocacy conference] in 20##. That’s where I actually met [child’s specialist]. He was speaking there… And I’m a little shy, so <unintelligible> I walked up to him and I said, you know, ‘Are you taking new patients?’ And I almost made an appointment right there. So, that was how we connected with him and we’ve been very happy with [hospital’s] team.*” (116000)

“*The best advice I can give is make sure you find a doctor that will listen to you… She could look at him and tell, like, “Okay, something’s not right.” Or she can smell him and tell that something’s not right…-- just know your kid*.” (150001)

Family caregivers’ interviews reveal that often, building an optimal care team is impacted by prior experiences with healthcare providers who have limited knowledge of treating patients with rare conditions. In some cases, such experiences are noted to have resulted in negative health outcomes due to medical crises. In other cases, narratives of such experiences seem to dehumanize the child. In all, how the medical community interacts with patients and families affected by rare NBS-related conditions shapes caregivers’ perceptions of the health care system—including feelings of medical mistrust—adding further complexity to building an effective care team for the child and family.

“*I think like a big thing with me is dealing with, like, trust with doctors because when [child] was diagnosed, the doctor said, ‘He doesn’t have [condition]. There’s no way he has [condition]. It’s too rare of a disease…Like he—you know, he doesn’t have it.’ So then he ended up having it. So now, like, if, you know, there’s a health issue that arises and the doctors say we don’t have it, I don’t believe them and I don’t trust them, you know.*” (119000)

“*He’s a person with feelings and experiences and day-to-day life, you know, that have to be taken into consideration. And they just kind of view him as, like, ‘Wow, this is exciting. Like, once in a lifetime we get to see one like this.’ You know, stuff like that. We’ve had people—doctors—say that to us. And I find that to be very offensive and (laughs) detrimental to the relationship…But because they had no ability to care for him personally, you know, we kind of eliminated them from the repertoire of people we had to see… And I always have thought here that they’re goal is not just to, you know, learn about this science experiment, but they’re goal is to give [child] the best quality of life he can have.*” (141000)

While healthcare professionals and caregivers strive to bring their knowledge, experience, and unique skillsets to the table and are mindful of the best interests of the child, their ability to optimally work together can be significantly influenced by how they communicate with each other.

“*…trying to be assertive, as well as respectful, and get heard, like, can be really hard things when you’re dealing with, like, the medical community and you don’t have any kind of degree you feel, like—and you’re, also, just trying to learn it through, like, a psychosocial component of that, too. Because different medical doctors have different personalities and, like, different triggers, right? Like, if I say something one way to one doctor, it’s going to rub him the wrong way. And, then, he has a perception of me. And, then, like, I don’t know. Like, you hope that they hold you with as much grace as you try to hold with them.*” (747000)

#### 3.1.5. “Unfortunately, the situation isn’t such what … we have this treatment and that treatment. It’s so specific and so specific to our own family…” (700001)

For many of these caregivers, uncertainty related to the child’s health outcomes was depicted as being inextricably tied to challenges with disease management and treatment options, which are often limited and individualized due to the rarity of such conditions and the phenotypic variation expressed within specific diagnostic categories. These factors present further complexity as caregivers navigate an expanding healthcare system due to the evolution of the condition, diagnosis, or therapeutics, each impacting care decisions for their child that must be (re)evaluated.

“*… So, I called Dr. [X], and I said, ‘What do we do? What are our options?’ He said, ‘Well, we could do a liver and kidney transplant when he’s about three months old.’ And at that time, and still, kind of still actually, there’s still lots of problems that go on with transplants. You don’t just give someone a transplant, and they live 80 years, and they’re perfectly fine. I mean, you do have stories like that. Don’t get me wrong. But when you have [condition], there’s still other problems that are associated with these kids, so it doesn’t just all of a sudden, rainbows come out and the sun’s shining and you don’t have any more problems. It’s not like that.*” (100000)

Caregivers are often called upon to make difficult and emergent decisions as they experience the push and pull of balancing potentially invasive interventions and their child’s quality of life, especially when information is limited or outcomes are unknown. To help them make informed decisions—decisions that are often described as temporally challenging—caregivers speak of needing to increase their literacy regarding the rare disease (often through their lived experience) as well as the potential interventions or treatment(s) for their child while building a knowledgeable care team. However, doing so (even when they have a medically named diagnosis) does not always lead to a clear understanding of the child’s prognosis, care, and treatment or prevent deterioration in health outcomes.

“*I mean, when he was age zero to three, before that liver transplant, every day was, like, brutal. He was so volatile from a health perspective …in hindsight, I think we thought, at the time, the G tube represented failure to some degree. But I think as time went on, and his issues became more clear, and the reality of things became more clear, you know, we kind of pulled the ripcord and got that G tube put in …We probably should have done that sooner, but, you know, you live and learn or whatever. And then, the liver just changed everything…I mean, he, you know, we were at the shore, you know, watching him run down the boardwalk, you know, and unfortunately, that stroke robbed him of that kind of stuff. And you know, the biggest—the shame of it—of everything he went through and all the issues he faces and that damn stroke, like, robbed him of, and created challenges for him on a day-to-day basis that don’t seem like they’re going to be able to be significantly mitigated.*” (135001)

The interviews also highlight that new genomic technologies offer these caregivers hope that such treatment interventions, and associated decisions, are just around the corner or at least on the horizon.

“*It’s not like it’s something that everybody has and they’re going to be looking—…for a cure. It’s the way that you’re going to solve this disease--the only way you’re going to cure this is that there’s going to be some—somebody’s going to come with some breakthrough in like genetic sequencing or something or being able to change, you know, change the genome around, or you know, make those kind of changes that can be generic for every kind of disease.*” (116001)

“*We are very positive…We think that, “Okay, this will work out.’ He have a [sic] deafness, we got cochlear implant for him. He’s losing his vision. We are hoping, and we are very positive, that someday they will come up with something where his vision problem will be solved. Some genetic modification or some gene therapy or something.*” (134001)

### 3.2. Domain 2. Psychosocial Implications of a Child Diagnosed with NBS-Related Condition: “We just don’t even have any clue what the outcome’s going to be like in this.” (116001)

In addition to navigating the child’s medical care, these caregivers report that they are also coping with the myriad of intricacies when caring for a potentially medically fragile child where the outcomes may be uncertain. As one caregiver stated:

“*And when you have a child with an inborn error of metabolism, every day is unknown. There are worst case scenarios that are very real, and possible, and even sometimes probable.*” (722000)

Indeed, the caregivers point to the fact that—“everybody develops different” (115000)—and this variability was noted to have a broad impact on these caregivers’ experiences. Importantly, as caregivers connect with each other, they spoke of gaining an understanding of this variation and developing a support system that they were able to leverage as they navigated their caregiving role:

“*But it was a relief many ways to meet other families whose children were not severely—I mean, there were some who had more severe parents [sic patients] but some whose kids seemed pretty normal, so that was a lot better than, you know, what the doctors that we currently had, the picture they were painting.*” (116000)

“*I went to some parent group when he was in—early on, and they were like, [the child] will—he will do his own thing. It will be his own path, and there’s no—there are no marks for this road. You don’t have any benchmarks…It’s just a different perspective once you recognize he won’t ever fit in this normal box, so it’s kind of freeing that way.*” (149000)

The uncertainty, both in terms of how the condition manifests symptomatically for the child, as well as for the child’s future, also impacts family caregivers’ psychosocial adjustment. Caregivers’ reports describe adjustments related to integrating the child’s diagnosis and medical management into the caregiver’s day to day activities; the process of acceptance, which requires them to adjust their perceptions and feelings about the child’s abilities and future outlook; and how caregivers plan for an uncertain future, both for themselves and the child.

#### 3.2.1. “…We’re kind of in a battle rhythm…we know for the most part what we’re dealing with.” (126001)

Following a presumptive diagnosis of an NBS-related condition, many of these caregivers describe learning a new “battle rhythm” in an effort towards integrating disease management into everyday life. Caregivers report that although the day-to-day activities associated with disease management initially overtake their lives, they are able to adjust to, redefine, and create their ‘new normal’. Narratives show that this adjustment may be in the form of feeling capable to address a crisis that may arise, and through gaining a different perspective with respect to what it means to have a good enough or an optimal quality of life:

“*Getting used to just kind of living with the uncertainty, and living with that, you know, being ready to, you know, change tactic at a moment’s notice.*” (138001)

“*And try to adjust your life. You don’t have to live in a cave and not travel anywhere and, you know, -- this family said the doctors told us that, you know, she needs to stay home and not leave because if it gets too cold they could die. Well, yeah, they can. But is it worth living your life that way? …For us, you know, [the child] needs to ride rollercoasters. So what if she shouldn’t because she’s got problems with her heart and stuff? If she died on a rollercoaster, that would be great because she would go out happy.*” (131000)

#### 3.2.2. “And you learn to appreciate what you have and not what you don’t” (126001)

Adjustments in the caregiver’s roles are reflected in daily activities, and also in their perceptions, feelings, and expectations as expressed in the interviews. They tell stories whereby this coping process involves acknowledging the feelings, good and bad, that come with caring for a child with an NBS-related condition, and accepting what one can control and what one can’t:

“*And actually, life becomes normal and a lot of the time you don’t think about it. When you get used to it it’s just your life and our life is normal. And you have feelings in the beginning that you shouldn’t be afraid of, to express. If you feel like you wish you wouldn’t have had that child, allow yourself to feel it. Allow yourself to feel everything because you have to work through all those feelings.*” (107000)

“*people need to be as educated as they can about the disease process so that they can try to minimize any problems. But then, at the same time, you have to embrace that there’s a lot of things outside of our control.*” (127000)

“*And you know, certainly, I’ve accepted some realities over time, and adjusted what I thought ‘good’ looked like.*” (135001)

#### 3.2.3. “fear of the unknown, fear of the unknown future” (119001)

While caregivers shared stories across a continuum of hope, optimism, and expectations that new discoveries will allow the child seriously affected by the NBS-related rare condition to live independently, they also reported on the psychological toll related to their fears of the future and the process of acceptance:

*“-- you kind of have this whole future planned out in your head for them, and then, when you get this diagnosis, it’s just like—almost like a punch in the stomach. Like, you don’t—everything becomes uncertain, unstable, and it’s very—I mean, it’s heartbreaking.”* (740000)

“*-- you don’t know when something bad can happen, so I’m always thinking the worst. I don’t really know. Like, I don’t—I don’t know if my kids are going to go to college. I don’t know if my kids are going to, like, have families.*” (146000)

“*I think when [child] was younger I had so many goals and so many, I don’t know, things in mind for him or hopes and stuff. And it was so painful when, I guess, things kind of would slowly but—it’s interesting because I think when he was really tiny, like a little baby, I think part of me already knew how bad things were going to be at some point. And I didn’t want to like accept that, so I kept hoping things were going to change or get better, or he wouldn’t be as bad as I guess I thought. And I think it has turned out to be as bad as I feared (laughs)—very early on. But, I don’t know, I feel like that’s almost part of the coping that you like—you come to accept that little by little, rather than just—because it’s too much. Like it’s too overwhelming to try to accept the depth of how bad things will be. Like at—all at once, you know?*” (141000)

## 4. Discussion

Narratives from retrospective in-depth interviews with this relatively large sample of family caregivers of children with NBS-related conditions provide rich empirical data describing their evolving journey and the challenges they experience as they navigate a landscape replete with unknowns, uncertainty, and variability. These caregiver stories contribute valuable ‘lessons learned’ that can inform ethically minded integration of genomics within a population-based newborn public health program—implementation that fosters respect for informed shared decision making—by bringing to light a constellation of challenges that largely fall within two domains. The first domain focuses on their critical caregiving role that necessitates developing medical expertise, advocating for the child with the rare condition, and navigating the healthcare system; whilst the second highlights psychosocial aspects of adjusting to and coping with their caregiving role. These domains reflect both problem-focused coping as they attempt to manage their child’s condition and emotion-focused coping as caregivers realign their expectations of what is important for their own and their child’s lives.

Given the inherent characteristics of the majority of NBS-related conditions—notable phenotypic variability, prognostic uncertainty, and a wide range of gene variants with low phenotypic correlation in concert with current limited knowledge of rare diseases by health care professionals and others—we anticipate that many of the significant challenges expressed by these family caregivers will persist for other families as more conditions are identified with population-based genome approaches. We acknowledge that the challenges identified here are not all new and there are prior and current efforts to address them. However, many of the challenges raised by family caregivers in these interviews highlight that they have not yet been sufficiently met. Thus, there is a very real need to further mitigate the challenges that caregivers of children with rare diseases experience in order for a more sequence-informed NBS to be realized, including complementary to biochemical assessments. Based on the domains and themes identified from the narratives and perspectives of these family caregivers, we share several points of consideration to optimize ethically nuanced NBS expansion augmented with sequencing, with the hope that such lessons learned can potentially further inform policy recommendations for the benefit of the child, the family, the healthcare system, and society.

### 4.1. Domain 1. Medical Management Implications of a Child Diagnosed with NBS-Related Condition

The provision of up-to-date enhanced **educational resources for pediatricians and other primary healthcare professionals (and students)** regarding the increasing number of rare conditions that are potentially NBS-identifiable with genomic technologies will be essential to reduce incorrect assumptions of false-positive results and minimize the expressed negative impact when a presumptive diagnosis is initially communicated to families. In addition, there is a need to educate healthcare professionals about the screening to the diagnostic process and address incorrect assumptions about screening and confirmatory testing, including realistic expectations of repeat screening and the speed of results. This in turn may help alter the experience of losing trust in the healthcare system while on their diagnostic odyssey and beyond, including understanding their child’s condition and seeking treatment.

Readily accessible and appropriately vetted **educational resources for families** regarding rare conditions that are potentially NBS identifiable with genomic technologies is an imperative to minimize the expressed negative impact when a presumptive diagnosis is communicated to families and thus help alter the experience on their diagnostic odyssey. We recommend provisions of responsibly vetted educational resources, including a general web link to help families locate IRB-approved government registered clinical trials and designated centers of excellence and/or clinical researchers recognized to have expertise in this specialized space.

Empowerment programs that help **family caregivers build capabilities** to advocate for the child, both in terms of treatment and intervention, but also to improve quality of life. Caregivers will take on the role of patient advocate for the child, and thus building communication and advocacy skills, along with literacy, early in the caregiving career will help caregivers interface with the healthcare system and improve treatment and care provided to the child. Due to the range of experiences, baseline literacy, and time constraints that caregivers have, it is imperative that these programs be developed and implemented through an equity and access lens.

Training in the principles and practice of **patient-centered communication** [48,49,50] **for providers**, which has long been identified as a fundamental physician competency. Components of patient-centered communication include (1) responding to emotions; (2) managing uncertainty; (3) enabling disease- and treatment-related behaviors; (4) exchanging information; (5) shared decision making; and (6) fostering healing relationships [51]. Improved patient–provider communication can help to build trust, and in turn, reinforce caregivers’ capabilities to advocate and care for the child.

Ease of access to **multi-disciplinary specialists**, both in person and virtual/remote, to help with the diagnostic odyssey and acute and chronic long-term care management, and to improve more realistic understanding and expectation of the condition as its manifestations evolve. Equitable access to specialist care and advice may have a positive impact not only on the child’s health but may also help families adjust to shifting ‘new normal’ and plans for the future.

### 4.2. Domain 2. Psychosocial Implications of a Child Diagnosed with NBS-Related Condition

The affordable and accessible provision of **continuous, long-term counseling resources** (genetic counseling, psychology, social work) for families to support, better understand, and cope with the diagnostic odyssey continuum and the evolution of the child’s condition as increasing numbers of rare disorders become NBS identifiable with genomic technologies. These resources will be essential to minimize the expressed negative impact when a presumptive diagnosis is communicated to families and play an integral role to assuage the experience of adjusting to the “whirlwind” of their child’s condition and the evolving caregiver roles during the diagnostic odyssey continuum.

Accessible **patient navigator resources and complex care programs with transition services** for families who have children with rare conditions that are potentially NBS identifiable using genomic technologies. This is imperative to minimize the expressed negative impact of attempting to navigate healthcare systems and manage conditions after a presumptive diagnosis is communicated to families and thus help alter the experience beyond the initial diagnostic odyssey.

**Peer support programs**, including mentoring programs facilitated by clinicians trained in psychosocial services, that connect families with shared experiences. Such programs provide an opportunity for caregivers whose children have relatively similar needs and/or prognoses to learn from each other and foster support for each other as they navigate psychosocial challenges related to the uncertainty surrounding the child’s diagnosis, disease course, and potential therapeutics [52].

Keeping in mind the two domains described here, it is important to consider that they are intertwined. With a lens towards ethical tenets of non-maleficence, beneficence, autonomy, and justice/fairness [53] for the family as well as the infant/child, this data from psychosocial research offers considerations that emphasize respecting key stakeholder informed decision making and empathically managing expectations related to prognostic certainty, outcome, quality of life, and care. Central to these considerations is an ethical framework grounded in the harm principle [35] and the best interests of the child [33,34]. Care team interactions can be sources of stress and strain for caregivers, thereby impacting caregivers’ psychosocial well-being and the well-being of the child. Thus, identifying and implementing best practices to help families navigate the healthcare system can alleviate some of the associated strain. Provider interactions that are empathic and bi-directional exchanges provide an opportunity for open communications such that healthcare providers and caregivers can learn from each other and be equal partners in care decisions for the child. For example, providers who are empathically direct about the assumptions underlying diagnostic testing and the length of the diagnostic odyssey help families gain knowledge and understanding about the process. Moreover, caregivers who feel comfortable openly discussing their concerns and experiences can educate providers to fully appreciate what it means to care for a child with a rare condition.

Fundamentally, such practices demonstrate respect for persons, a key characteristic of the principle of autonomy, and shared decision making, particularly when children are involved [25]. These ethical considerations can, and should, be addressed through the provision of improved education, communication, support, and equitable access from before the screening process to the initial diagnostic label to evolving symptomatology, care, treatment, and future planning for all involved. “So long as the child is part of a viable family, his [sic] own interests are merged with those of the other members” [33]. With this acknowledgment, the voices of family caregivers in this study resonate and deserve to be heard, with strategies implemented to address their concerns as genomic expansion evolves within population-based NBS. To do less is ethically unjustifiable.

## 5. Limitations

As the promise of genomic integration into NBS becomes realized, it is important to consider the experiences of caregivers in informing best practices for translation into a population-based screening program, and future research should include a more broadly diverse group of caregivers than the scope this study could offer. Here, we provide recommendations that stem from the narratives provided by caregivers of children with NBS-related conditions. Importantly, those who provided their perspective through in-depth qualitative interviews were referred to this research through ongoing natural history protocols at the National Institutes of Health and through related advocacy groups. Participants largely identified as non-Hispanic and White and had completed post-secondary education. The majority of children receiving care were diagnosed with a number of inborn metabolic disorders. Thus, the results herein should be interpreted with this in mind and may not be generalizable to the rare disease community as a whole or to the general population for which NBS as a public health measure serves. Despite this, these findings are based on a large sample, and caregivers provided rich narratives regarding their journey through the diagnostic odyssey continuum with themes that other caregivers have expressed, and others may experience. Finally, the scope of ethical considerations and NBS details considered here were purposefully limited in accordance with this Special Issue’s aim to minimize redundancy of the ethical-psychosocial aspects raised and the associated scholarship presented within this collection.

## 6. Conclusions

The promise of expanding population-based newborn screening to include ever more increasing degrees of molecular sequencing may be realized in the near future. For some, these new technologies may shorten the diagnostic odyssey and may lessen the diagnostic odyssey continuum, whereas for others there may be a lengthier diagnostic odyssey continuum due to greater identification of conditions that may never manifest as disease. However, in either case, greater integration of genomics into public health NBS programs will likely not change most of the experiences reported herein without multi-faceted efforts to seriously address these challenges and the ethical considerations raised.

## Figures and Tables

**Table 1:** Caregiver and child socio-demographic characteristics.

Participant Characteristics (*N* = 169)		M (SD) or % (*N*)
Gender		
	Female	66.9% (*N* = 113)
	Male	33.1% (*N* = 56)
Age		M = 43.3 years (SD = 12.7)
Marital status		
	Married/living as married	82.2% (*N* = 139)
	Never married	7.1% (*N* = 12)
	Divorced or separated	5.3% (*N* = 9)
	Widowed	0.6% (*N* = 1)
	NA	4.7% (*N* = 8)
Kinship		
	Mother	44.4% (*N* = 75)
	Father	26% (*N* = 44)
	Sibling	3.6% (*N* = 6)
	Grandparent	14.2% (*N* = 24)
	Aunt/uncle	5.9% (*N* = 10)
	Other kin	1.2% (*N* = 2)
	Non-kin	4.7% (*N* = 8)
Education Level		
	Middle school/some high school	1.2% (*N* = 2)
	High school graduate/GED	10.1% (*N* = 17)
	Associate degree/technical training	22.5% (*N* = 38)
	Bachelor degree	42.6% (*N* = 72)
	Post-graduate degree	20.7% (*N* = 35)
	NA	3.0% (*N* = 5)
Income Level		
	>USD 200,000	11.8% (*N* = 20)
	USD 100,001 to USD 200,000	28.4% (*N* = 48)
	USD 50,000 to USD 100,000	29.6% (*N* = 50)
	USD 20,001 to USD 50,000	16.0%(*N* = 27)
	<USD 20,000	4.8% (*N* = 8)
	Don’t know	5.8% (*N* = 10)
	NA	3.6% (*N* = 6)
Race		
	White	84% (*N* = 142)
	Black or African-American	3.5% (*N* = 6)
	Asian	3.0% (*N* = 5)
	Mixed race	3.0% (*N* = 5)
	Other	4.1% (*N* = 7)
	NA	2.4% (*N* = 4)
Ethnicity		
	Hispanic or Latino	5.3% (*N* = 9)
	Non-Hispanic or Latino	90.0% (*N* = 152)
	NA	4.7% (*N* = 8)
	**Child Characteristics (N = 77)**	**M (SD) or % (*N*)**
Gender		
	Female	36.4% (*N* = 28)
	Male	63.6% (*N* = 49)
Age		M = 10.3 years (SD = 7.8 years)
Diagnosis Category		
	Organic acidemia	80.5% (*N* = 62)
	Fatty acid oxidation disorder	5.2% (*N* = 4)
	Carbohydrate metabolism disorder	1.3% (*N* = 1)
	Lipid storage disorder	3.9% (*N* = 3)
	Mitochondrial disorder	3.9% (*N* = 3)
	Undiagnosed condition	5.2% (*N* = 4)
Childhood Activities of Daily Living		
	Life skills [Range: 0–20]	M = 12.9 (SD = 6.7)
	Social skills [Range: 0–16]	M = 7.6 (SD = 5.5)
	Behavioral [Range: 0–24]	M = 3.3 (SD = 4.0)

## Data Availability

The data that support the findings of this study are available from the corresponding author upon reasonable request.

## Supplementary Materials

The Supplementary Materials is available online at https://www.mdpi.com/article/10.3390/ijns8020035/s1.
