# Supplementary material for: Experiences of Families Caring for Children with Newborn Screening-Related Conditions: Implications for the Expansion of Genomics in Population-Based Neonatal Public Health Programs
Source: Int J Neonatal Screen. 2022 May 23;8(2):35. doi: 10.3390/ijns8020035 (PMC9149923; doi:10.3390/ijns8020035)
Supplement: Supplementary file 1 [file IJNS-08-00035-s001.zip › IJNS-1681178-supplementary.pdf]

Open-ended Interview Guide

Adapted from: Murphy NA, Christian B, Caplin DA, Young PC. The health of caregivers for children with disabilities: caregiver perspectives. Child Care Health Dev. 2007;33(2):180-187.

1. How does your family balance the needs of [affected relative's name] and the needs of other family members?

Parenting and caregiving can be stressful, and people use many different coping strategies to deal with their responsibilities, their circumstances and other sources of stress. The following two questions are asking about your family's coping strategies.

2. What do you appreciate about the way that your family works?
   -How has your family member's illness influenced your family in a positive way?
   -What has worked well for you as a family in coping with the illness?
3. What do you wish would change in your family?
   -What coping strategies has your family used that might not have been effective?
4. Describe what a typical day looks like in your home.

5. How does being a caregiver for a person with a chronic illness affect your physical health?

6. Are you doing anything to maintain your physical health?

7. Are you doing anything to maintain your mental/emotional health?

8. What are the different emotions you experience in this role as a caregiver?

9. Do you talk to your doctor or mental health professionals about your emotional health? Do you discuss feelings of depression, anxiety, or guilt? Do you receive treatment for these feelings?

10. Have you heard of the term "burnout"? What is "burnout" for you? Do you experience caregiver "burnout"? How often?

11. What do you do to take care of yourself during periods of high demands and high stress? Are you able to take a break when you feel as though you need one?

12. Do you get regular respite or a regular break every now and then from caregiving? How often do you get these breaks?

13. Do you receive services from community or government social service organizations? If so, what type of services or resources do you receive from where? How often do you receive them? Do you feel that you receive enough help? What other services would help you?

14. What goals do you have for your family's future? How do you envision your family's future?

15. What advice would you give to another family who has a member facing the same condition?

16. What was it like for you to talk with me about these things?

17. Is there anything else that we haven't talked about that you would like to speak about regarding how the caregiving process has affected you?
